# Surface Modifications of Poly(Ether Ether Ketone) via Polymerization Methods—Current Status and Future Prospects

**DOI:** 10.3390/ma13040999

**Published:** 2020-02-23

**Authors:** Monika Flejszar, Paweł Chmielarz

**Affiliations:** Department of Physical Chemistry, Faculty of Chemistry, Rzeszow University of Technology, Al. Powstańców Warszawy 6, 35-959 Rzeszów, Poland; d442@stud.prz.edu.pl

**Keywords:** PEEK, surface modification, polymer brushes, ultraviolet (UV)-initiated graft polymerization, SI-ATRP

## Abstract

Surface modification of poly(ether ether ketone) (PEEK) aimed at applying it as a bone implant material aroused the unflagging interest of the research community. In view of the development of implantology and the growing demand for new biomaterials, increasing biocompatibility and improving osseointegration are becoming the primary goals of PEEK surface modifications. The main aim of this review is to summarize the use of polymerization methods and various monomers applied for surface modification of PEEK to increase its bioactivity, which is a critical factor for successful applications of biomedical materials. In addition, the future directions of PEEK surface modifications are suggested, pointing to low-ppm surface-initiated atom transfer radical polymerization (SI-ATRP) as a method with unexplored capacity for flat surface modifications.

## 1. Introduction

Currently, the production of bone implants is limited only to metal materials (stainless steel, cobalt–chromium, titanium). However, in the production of personalized bone implants, there is an alternative synthetic polymer named poly(ether ether ketone) (PEEK) [1,2,3]. The chemical structure of PEEK can be defined as an alternating combination of aryl rings through ketone and ether groups; therefore, it belongs to the family of polyaryletherketone polymers. The Yang’s modulus of pure PEEK is close to the elastic modulus of the bones and is about 3.6 GPa, which is an unquestionable advantage of this material over ceramic or pure steel implants [4]. Furthermore, PEEK has high abrasion resistance, low friction coefficient, and low sensitivity to temperature change [5]. The glass transition temperature of amorphous PEEK is about 145 °C, and the melting temperature of pristine polymer is about 343 °C [6]. The synthetic process of obtaining PEEK material is based on the dialkylation of bisphenolate salts, which results in a chemically resistant, non-biodegradable product. In the case of the production of permanent implants, the lack of biodegradability is a desired feature and determines the usefulness of the material. Therefore, since the United States (US) Food and Drug Administration (FDA) certified PEEK as a suitable bone implant material in the 1990s, the application potential of the material in the orthopedic engineering is constantly increasing [7].

Implants made from poly(ether ether ketone) can be used in complicated reconstructions, even in areas with difficult access [8]. The fastening of an implant to surrounding tissues can be performed by using standard screws chosen by a surgeon. Moreover, PEEK implants are transparent to X-rays, which is a very beneficial factor in the analysis of treatment progress by computed tomography or nuclear magnetic resonance in the postoperative recovery phase. Additionally, PEEK can be sterilized with popular methods such as the use of steam under pressure, gamma radiation, or ethylene oxide. 

Typically, the manufacturing of an implant made from PEEK is based on structuring it by milling from the block of material. Due to its rather high hydrophobicity, it shows good bioinert properties, but not sufficient ingrowth in the surrounding tissue. This is the reason why it is mainly used for injuries where no shear forces will act on the surgery site, for example, with screw-fixated vertebral bodies in the spine. Therefore, there is no real adhesion between the implant and surrounding tissue, as it is only tightly surrounded by the freshly formed bone tissue [9].

The above-mentioned problem with the adhesion of living tissue with an implant made of PEEK drives scientists to search for effective methods and chemicals to modify the surface in order to improve its bioactivity. Among the methods for surface modification of the implant material, a number of mechanical, chemical, or physical methods can be distinguished. The high application potential of PEEK as a bone implant material intensified the research over the last decade, where various techniques of PEEK surface modification were described [10,11,12,13,14].

In modern times, surface functionalization is a driving tool for the development of new biomaterials with improved biological properties. Surface functionalization of inorganic and organic materials (e.g., PEEK) is mainly based on the absorption of substances, reaction with small molecules, or just grafting of polymer chains on the substrate surface, as presented in this review paper.

The application of controlled radical polymerization for surface modification of materials is based on the formation of covalent bonds between the modified surface and synthesized polymer brushes. In general, there are three synthetic approaches that enable the organic polymer layer to be permanently bonded to the modified material. The first approach called “*grafting from*” uses a previously functionalized surface with a covalently attached polymerization initiator containing a halogenated atom [15]. In the second approach, “*grafting onto*”, functional groups on the surface of the functionalized material are compatible with the chain-end groups of polymer brushes, resulting in the covalent attachment of the polymer chains [16]. In turn, “*grafting through*” is a two-stage process. At first, a polymerized monomer unit is attached to the modified surface; then, the created macromonomer is subjected to (co)polymerization with single monomer units [17]. The advantages and disadvantages of each of the mentioned synthetic approaches were pointed out in our previous paper about the creations of hybrid nanomaterials via surface-initiated atom transfer radical polymerization (SI-ATRP) [18,19].

The main objective of this review is to summarize the role of the polymerization process in the modification of the PEEK surface. The article presents the effects of using the traditional photo-induced polymerization process and classical surface-initiated atom transfer radical polymerization (SI-ATRP) to enhance the bioactivity of the PEEK surface, additionally indicating new possibilities offered by the application of the low-ppm or metal-free ATRP approach.

## 2. Photo-Induced Polymerization for Surface Modification of PEEK

**Figure 1:**
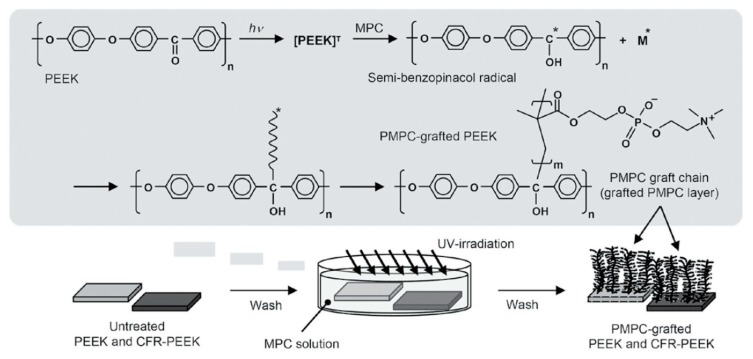
The scheme of poly(ether ether ketone) (PEEK) surface modifications via ultraviolet (UV)-induced polymerization of 2-methacryloyloxyethyl phosphorylcholine (MPC). Reprinted with permission from Reference [21].

Low cost, no complicated equipment, easy application in industry, and mild reaction conditions are just a few of the advantages that make photo-induced polymerization outweigh plasma-induced or radiation-induced polymerization. In 2009, Kyomoto and Ishihara [20] put forward hypotheses that the diphenylketone group present in the PEEK backbone acts as a photoinitiator in polymerization reactions, similar to benzophenone previously described in the literature. Self-induced grafting of polymer chains is powered by the formation of semibenzopinacol-containing radicals of benzophenone units in the PEEK molecular structure, as shown in Figure 1. The successful polymerization confirmed the assumptions and paved the way for the use of ultraviolet (UV)-initiated polymerization in PEEK surface treatment. The list of PEEK surface modifications carried out with the usage of photo-initiated polymerization is summarized in Table 1.

The first papers (2009, 2010, and 2013) on the successful self-initiated surface graft polymerization of 2-methacryloyloxyethyl phosphorylcholine (MPC) on PEEK by photoirradiation were published by Kyomoto [10,20,22]. A variety of performed tests revealed significant improvements in the water wettability, frictional properties, and wear resistance of modified PEEK surfaces. Moreover, the impact of the monomer concentration and UV exposure time on the extent of the grafted PMPC layer was investigated. The result showed that the grafting density and thickness of the polymer layer can be controlled by adjusting the concentration of the monomer and the length of irradiation time [10]. Furthermore, as an application to improving the durability of artificial hips, the nanometer-scale photo-induced grafting of poly(2-methacryloyloxyethyl phosphorylcholine) (PMPC) onto PEEK and carbon-fiber-reinforced PEEK orthopedic bearing surfaces and interfaces was demonstrated.

The continuation of research conducted by Ishihara et al. resulted in the publication of an article presenting the biological performance of the PMPC-grafted PEEK and its comparison with the unmodified PEEK surface [24,25]. It was concluded that an increase in the thickness of the PMPC layer caused a significant decrease in the amount of fibrinogen adsorption in comparison with the results obtained in the case of unmodified PEEK. Moreover, bacterial adhesion was reduced dramatically on the PMPC-grafted PEEK, which means that the antithrombogenicity of potential bone implant material was improved.

A similar concept presenting a PEEK surface modified with methacryloyl-functionalized MPC polymers (MAMA) was described by Kawasaki in 2014 [23]. At first, poly(MPC-*co*-AEMA) (PMA) was synthesized by conventional free-radical polymerization using 2,2′-azobisisobutyronitrile as an initiator; then, in contrast to previous studies, 1-butanol solution containing the PMAMA was dropped onto the PEEK plates, and they were immediately exposed to UV light. Various protic solvents (e.g., water, methanol, ethanol) for making polymer solutions were tried, but they did not spread well on PEEK. Then, fibrinogen adsorption patterns of a polymer surface after contact with human plasma were observed using scanning electron microscopy (SEM) pictures. Results of the blood compatibility test indicated that an appropriate layer of PMAMA was immobilized on PEEK.

Subsequent studies were aimed at optimizing polymerization conditions and presented high-efficiency preparation of the PMPC layer on PEEK in the presence of inorganic salt additive [26]. It turned out that the addition of inorganic salt (LiCl, NaCl, KCl) increases the polymerization rate and it may play a significant role by concentrating MPC in the solution through ionic hydration.

**Figure 2:**
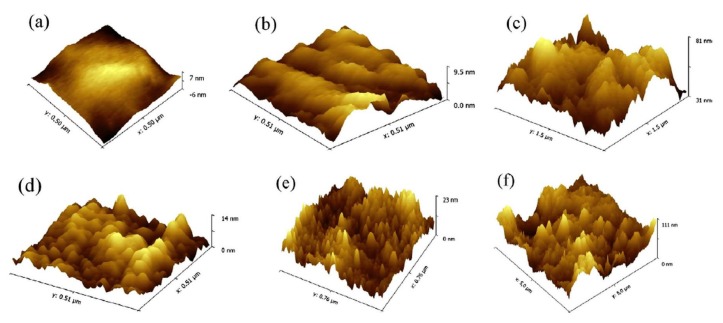
Atomic force microscopy (AFM) images of (**a**) unmodified PEEK sheet, (**b**) PEEK-*g*-poly(oligo(ethylene glycol) methacrylate) (PMeOEGMA), (**c**) PEEK-*g*-poly(acrylic acid) (PAA), (**d**) PEEK-*g*-poly(*n*-butyl acrylate) (PBA), (**e**) PEEK-*g*-polystyrene (PS), and (**f**) PEEK-*g*-poly(vinyl phosphonic acid) (PVPA). Reprinted with permission from Reference [27], 2014 Elsevier B.V.

The next step in the development of PEEK surface modification using photopolymerization was the grafting of polyelectrolyte brushes (2-(methacryloyloxy) ethyltrimethylammonium chloride (MTAC), 3-sulfopropyl methacrylate potassium salt (SPMK)), providing excellent antifouling properties and a low friction coefficient under wet condition [28]. Such modification opened up a variety of PEEK surface design possibilities for novel applications in harsh working and water-lubricated conditions. In contrast to the modifications previously described in the literature, the polymerization was carried out at room temperature, providing even milder reaction conditions, which are undoubtedly an advantage of the surface modification approach used. Another benefit of carrying out room-temperature polymerization emphasized by Yameen et al. [27] is the avoidance of monomer polymerization or its crosslinking. In the work mentioned above, a UV-light-mediated single-step procedure was successfully used for a variety of monomers and polymers with completely different chemical natures (e.g., *n*-butyl acrylate (BA), styrene (St), vinyl phosphonic acid (VPA), oligo(ethylene glycol) methacrylate (MeOEGMA), and acrylic acid (AA)). Then, the surface modification of PEEK was effectively evaluated by atomic force microscopy (AFM) measurement (Figure 2).

The surface characteristics of natural cartilage are often an inspiration to search for new bone implant materials with improved biological properties. The brush-like structures present on the surface of cartilage are aimed at supporting stress and impact, as well as adsorbing synovial fluid, which has an influence on properties such as wettability, lubrication, and friction. Unmodified PEEK is a bioinert but hydrophobic material, which is an important limitation in its use for the production of artificial joints. Therefore, a change in wettability leading to increased surface bioactivity is the main purpose of conducted modifications. In addition, the formation of a hydrophilic layer on the surface of the PEEK leads to improved wear resistance for aqueous lubrication. Referring to those facts, AA-enriched PEEK surfaces were prepared via UV-initiated polymerization [29]. As an effect, a material with a lower contact angle value was obtained, which clearly indicates an increase in surface hydrophilicity.

**Figure 3:**
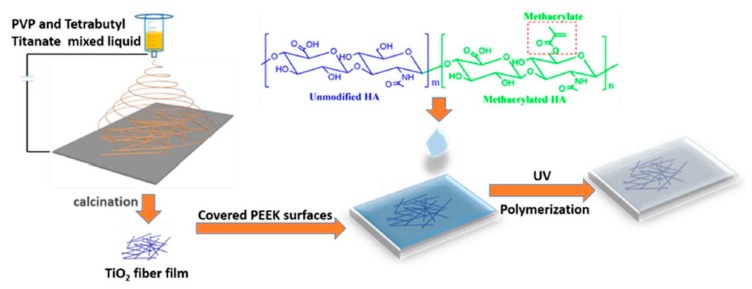
Schematically illustrated conception of PEEK surface modification based on light-inducible electrospinning. Reprinted with permission from Reference [30], 2018 Elsevier B. V.

Recently, bioactive PEEK, surface-modified simultaneously with electrospun titanium dioxide (TiO_2_) and methacrylated hyaluronic acid (MeHA) (PEEK-MeHA-TiO_2_) by ultraviolet irradiation, was created [30]. Similarly to the previously described modifications, the roughness and hydrophobicity of PEEK were greatly changed. With regard to properties of titanium dioxide (TiO_2_), which is especially well suited for functionalizing the material interface of medical devices, the authors presented conception based on light-inducible electrospun TiO_2_ that imitated the fiber topology of cortical bone, and they used it to fabricate a composite with PEEK (Figure 3). In turn, HA forms a polymer chain backbone with which mesenchymal stem cells (MSCs) can interact via receptors expressed on the cell surface; therefore, it was used to cover the external part of the created composite. As a result, the modified surface showed increased adhesion, proliferation, and osteogenic differentiation capacity.

More recently Zheng et al. [31] applied single-step ultraviolet-initiated graft polymerization for introduction of sulfonate groups onto the PEEK surface. The use of vinylsulfonic acid sodium (VSA) and vinylphosphonic acid (VPA) to improve the bioactivity of the treated PEEK surface opened a new modification path intensively applied in the latest research [31,32,33]. In vitro studies using the MC3T3-E1 line of osteoblasts showed that initial adhesion, spreading, proliferation, and osteogenic differentiation were greatly improved after PEEK surface modification. As proven in earlier studies, in comparison to hydrophobic surfaces, hydrophilic surfaces were more favorable for cell adhesion and proliferation [34]. Therefore, the surface-sulfonated PEEK implant material may improve bone–implant integration in comparison with pristine PEEK.

**Figure 4:**
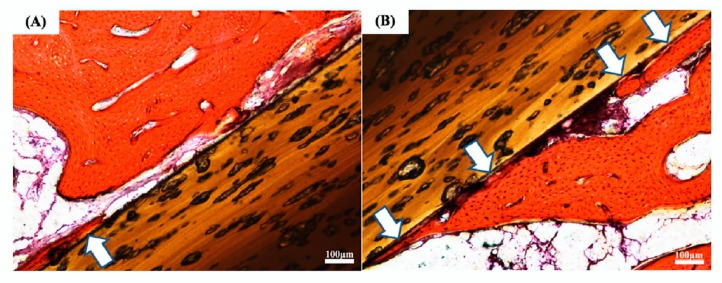
The photos were taken after 12 weeks of implantation in adult male New Zealand White rabbits. Direct contact between bone tissue and (**A**) pristine PEEK and (**B**) PEEK-*g*-PVPA is marked with white arrows. Reprinted with permission from Reference [33], 2019 Elsevier B. V.

The main conclusion confirming the validity of VPA modification to improve the bioactivity of PEEK was the result of tests carried out with an animal model (Figure 4). Higher bone–implant contact and a smaller space between the bone tissue and the surface-phosphorylated PEEK implants were observed. Interestingly, it was first shown by in vivo tests that PVPA had excellent bone tissue compatibility.

## 3. Surface Modification of PEEK via ATRP Method

ATRP is one of the most versatile techniques of reversible-deactivation radical polymerization (RDRP), which enables the synthesis of well-defined macromolecules with predefined structure, predetermined properties, and chain-end functionality [35,36,37,38]. In this case, the reaction mechanism is based on reversible activation and deactivation of propagating chains and catalyst complex molecules [39]. An equilibrium between active and dormant species is hardly shifted on the deactivation side, to maintain low active radical concentration, causing the equable growth of polymer chains [40,41]. In contrast to conventional free-radical polymerization, ATRP allows the extension of the lifetime of growing chains from seconds to hours or days [42]. To ensure the controlled nature of the process, the initiation stage is significantly faster than the propagation stage, which enables the simultaneous growth of all polymer chains. Moreover, control of the reaction is provided by an extension of the lifetime of propagating chains, which permits the synthesis of polymers with different topologies as well-defined block copolymers [43,44,45,46].

The application of ATRP in the synthesis of polymer brushes grafted from the flat surfaces creates the possibility of obtaining polymers characterized by narrow molecular weight distribution (MWD) and controlled architecture [47,48,49,50,51]. Additionally, SI-ATRP allows the growing functionality of polymer brushes from different surfaces [41,52,53,54,55] with a high degree of synthetic flexibility, enabling the introduction of a variety of functional groups [18,56,57]. Despite the high potential of SI-ATRP for the enhancement of PEEK surface properties, only limited research was conducted in this area. Nevertheless, this chapter summarizes the successful use of SI-ATRP for the modification of the bone–implant material. Surface modifications of poly(ether ether ketone) via the ATRP method are presented in Table 2.

**Figure 5:**
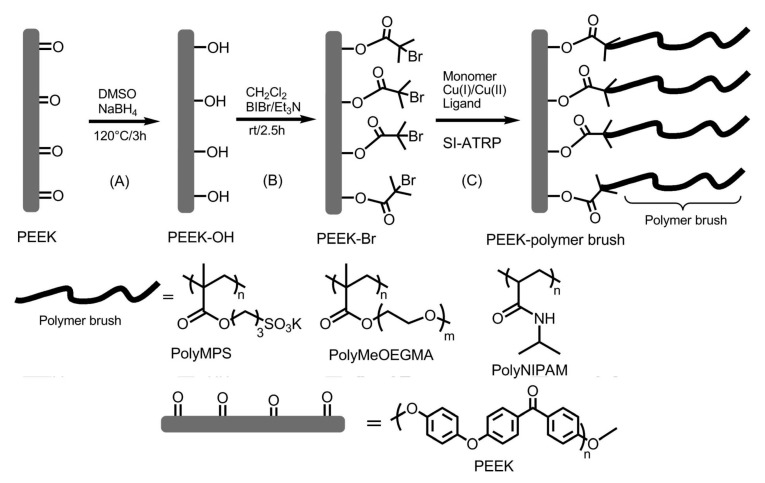
Schematic illustration of PEEK surface functionalization. (**A**) At first, keto group reduction was performed. (**B**) Then, atom transfer radical polymerization (ATRP) initiator (2-BiBr) was attached. (**C**) Subsequently, SI-ATRP of MPS, MeOEGMA, and *N*-isopropylacrylamide (NIPAM) was carried out. Reprinted with permission from Reference [58], ACS 2009.

For the first time, the unexplored capacity of SI-ATRP as a versatile methodology for controlling the surface properties of PEEK was presented by Yameen et al. [58]. Successful grafting of three different monomers, namely, potassium 3-(methacryloyloxy) propane-1-sulfonate (MPS), MeOEGMA, and *N*-isopropylacrylamide (NIPAM), was carried out according to Figure 5.

In the case of using the SI-ATRP approach, it is necessary to attach an initiator in the first step. For this purpose, the reduction of PEEK surface carbonyl groups to the hydroxy groups was performed, and then 2-bromoisobutyryl groups were covalently anchored at the PEEK surface as the polymerization initiator. Subsequently, for each of the grafted monomers, a different catalytic system was applied, i.e., 25,000 ppm of Cu^II^Cl_2_/2,2′-bipyridine (BPY) for MPS around, 2270 ppm of Cu^II^Br_2_/BPY for MeOEGA, and 10,000 ppm of Cu^II^Br_2_/*N*,*N*,*N*′,*N*″,*N*″-pentamethyldiethylenetriamine (PMDETA) for NIPAM. Successful PEEK surface modification was monitored by ATR infrared (IR) spectroscopy. The variety of properties of used monomers allowed examining the electrostatic interaction of PEEK-*g*-PMPS and rhodamine 6G, the antifouling evaluation performed for PEEK-*g*-PMeOEGMA, and the thermally responsive switching between hydrophilicity and hydrophobicity of PEEK-*g*-PNIPAM. From the implantology point of view, the most interesting result was presented for the PEEK-*g*-PMeOEGMA material. Surface modification was designed by the inspiration of materials coated with polyethylene glycol (PEG) or oligo(ethylene glycol), which prevent bioadhesion of proteins or living cells and bacteria [60]. The antifouling properties were demonstrated by exposing PEEK-*g*-PMeOEGMA to a culture medium of *Escherichia coli* bacteria. Interestingly, a significant number of bacteria attached to the surface of the pristine PEEK were observed, but there were no bacteria adhered to the PEEK-*g*-PMeOEGMA surface [58].

A similar approach with a significantly higher amount of catalytic complex (56,977 ppm of Cu^II^Cl_2_/BPY) was applied for the synthesis of PPEGMA on the PEEK surface via SI-ATRP. The concept of hydrophilization of bone–implant material using PPEGMA has a double benefit. Firstly, as in the examples described earlier, the hydrophilic layer causes the material to exhibit strongly limited protein adsorption. In addition, it allows for the deposition of a metallic layer, which, in the case of inert polymeric material, cannot be electrolessly metallized without proper surface treatment. In this case, one functionalization enables two different applications: electronics and medical [11].

## 4. Future Prospects and Conclusion

It is well known that surface characteristics and physico-chemical properties are key elements in modulating cell and tissue interactions with bone implant material. PEEK surface modifications can contribute to making it more favorable for human cell adhesion, growth, and differentiation, leading to enhanced implant–tissue integration. Over the past decade, two strategies (UV-initiated polymerization and traditional (high ppm) SI-ATRP) were mainly employed to improve PEEK surface biocompatibility.

A definite advantage of using the UV-initiated polymerization method for PEEK surface improvement is the one-step modification path. The lack of the need for additional actions related to the introduction of the initiator onto the modified surface makes this method simple and useful. Nevertheless, despite the successful application of UV-initiated polymerization, it still remains one of the methods of conventional radical polymerization not allowing for precise control of the architecture of the synthesized polymer. Thus, some limitations arise in the synthesis of, e.g., block (co)polymer coatings or gradient layers with extended topography. Therefore, the development of the UV-initiated technique in the context of flat surface modification is partially limited.

Despite numerous studies focusing on improving the biological properties of PEEK surfaces, there are still no published papers presenting the use of low-ppm RDRP solutions to improve the bioactivity of PEEK surfaces. Due to the fact that conventional ATRP requires a high catalyst concentration, leading to higher cost of purification of the final polymer product, low ppm ATRP techniques were proposed. In new ATRP methods, catalyst molecules are regenerated during the reaction progress [45,61,62,63,64]. There are many ways to achieve catalyst regeneration in ATRP, which can be distinguished as physical or chemical factors. Physical factors includes electrochemically mediated ATRP (*e*ATRP) [39,46,65,66,67,68,69,70], photo-initiated ATRP (π-ATRP) [71,72,73,74], mechanically induced ATRP (mechano-ATRP) [75,76,77], microwave-assisted ATRP (MW-assisted ATRP) [78,79], and novel ultrasonication-induced ATRP (sono-ATRP) [73,75,78,80,81]. Chemical factors include initiators for continuous activator regeneration (ICAR) ATRP [74,82,83], activator regeneration by electron transfer (ARGET) ATRP [84,85,86], and supplemental activator and reducing agent (SARA) ATRP [87,88,89,90].

Nevertheless, many low-ppm ATRP techniques possess some disadvantageous, such as a lack of control over the amount of copper present in the experimental system (SARA ATRP) or necessity for application of expensive devices (*e*ATRP). However, the ARGET ATRP approach eliminates the defects and provides simplification of the reaction system [91]. A potential advantage of this innovative solution is the possibility of a significant reduction in copper catalyst concentration (under 100 ppm), in comparison to the traditional ATRP reaction system. Furthermore, the application of ARGET ATRP enables carrying out the reaction under aerobic conditions [92]. Another profitable aspect is the opportunity for substantial process cost minimalization by elimination of expensive potentiostat devices according to the *e*ATRP approach [68]. The use of ascorbic acid as a reducing agent is one of the cheapest (cheaper than an ultrasonic cleaner (needed in sono-ATRP) and a typical electrochemical set-up) solutions leading to the reduction of the amount of catalytic complex. In addition, ascorbic acid is approved for use by the US FDA, which confirms the legitimacy of its use in the syntheses leading to the production of biomaterials (e.g., PEEK surface modifications) [93].

An additional convenience of the low-ppm ATRP approach is the resignation of organic solvent usage, due to the possibility of conducting the reaction in a miniemulsion system. The biphasic nature of water–oil dispersion simplifies catalyst removal, while also providing increased contact surface area and facilitating mass and heat transport. ATRP in a miniemulsion system is an environmentally friendly solution, as it requires water as solvent, which is less toxic and less expensive than most organic solvents [41].

On the other hand, contamination of even small amounts of inorganic catalysts in biological systems can be slightly problematic. However, a metal-free ATRP based on regeneration of the deactivator with application of UV light is a promising prospect for the future research in this area [94,95]. For this reason, the focus on the use of organic photo-reduced catalysts in ATRP reactions might open up a new doorway for the design of biologically compatible systems.

In view of the possibility of using a wide range of various monomers, ATRP with diminished catalyst concentration can be a highly versatile and powerful tool for controlling the surface properties of inorganic and organic materials (e.g., PEEK). Firstly, effective optimization of SI-ATRP can lead to an ultra-high thickness of polymer brushes grafted from the flat surface. Secondly, following the 12 principles of green chemistry, in order to design a more environmentally friendly experimental setup, the use of organic solvents can be reduced by performing syntheses in water or using a miniemulsion system. Moreover, the low concentration of the catalytic complex simplifies the purification of the final product. Additionally, the concept of grafting from a polymer also allows an initiator gradient, enabling variation in the grafting density, resulting in materials with increased functionality. Therefore, future work should focus on involving an attempt to graft biocompatible (co)polymer brushes from the PEEK surface via a low-ppm RDRP approach.

An ideal bone implant material remains to be developed; thus, there is an intensive need for research on alternative materials with improved biological properties. Only limited monomers were grafted from the PEEK surface; however, the composition of the surface of the material guaranteeing ideal conditions for the adhesion of the implant with bone tissue remains to be determined. Nevertheless, there are literature reports of positive results from in vitro and in vivo tests performed on PEEK modified via surface-initiated polymerization. The increase in the implant’s adhesion capacity with living tissue confirmed by in vivo tests shows that research in the right direction already started. Nevertheless, it is worthwhile to use a combination of various surface modification methods. At the same time, it should be noted that special emphasis is placed on carrying out synthetic procedures according to environmentally friendly principles. Therefore, modern methods of polymerization (e.g., light-induced controlled polymerization) can be a useful and versatile tool for the synthesis and modification of new biomaterials. Another challenge for future science is to find new biologically compatible monomers, which can be successfully polymerized with, e.g., the low-ppm SI-ATRP method, as well as metal-free controlled radical polymerization, leading to a breakthrough in the bone implant material industry.

## Abbreviations (Alphabetical Order)

AAacrylic acidAEMA2-aminoethyl methacrylate hydrochlorideAFMatomic force microscopyBA*n*-butyl acrylateBPY2,2′-bipyridineHAhyaluronic acidMAMApoly(MPC-*co*-AEMA-*co*-*N*-methacryloyl methacrylamide)MeHAmethacrylated hyaluronic acidMeOEGMAoligo(ethylene glycol) methacrylateMPC2-methacryloyloxyethyl phosphorylcholineMPSpotassium 3-(methacryloyloxy) propane-1-sulfonateMSCmesenchymal stem cellMTAC2-(methacryloyloxy) ethyltrimethylammonium chlorideNIPAM*N*-isopropylacrylamidePEEKpoly(ether ether ketone)PMApoly(MPC-*co*-AEMA)PMDETA*N*,*N*,*N*′,*N*″,*N*″-pentamethyldiethylenetriamineRDRPreversible-deactivation radical polymerizationSEMscanning electron microscopySI-ATRPsurface-initiated atom transfer radical polymerizationSPMK3-sulfopropyl methacrylate potassium saltStstyreneTiO_2_titanium dioxideUVultravioletVPAvinylphosphonic acidVSAvinylsulfonic acid sodium

## Figures and Tables

**Table 1:** Surface modifications of poly(ether ether ketone) via photo-induced polymerization method.

Monomer	Solvent	T ^1^ (°C)	Time (min)	Wavelength (nm)	Light Intensity (mW/cm^2^)	Reference
MPC	H_2_O	60	5–90	350 ± 50	5	[10,20,21,22] ^2^
MPC	1-butanol	NIA ^3^	NIA	350 ± 50	1	[23]
MPC	H_2_O	25–60	90	350 ± 50	1.5–9	[24]
MPC	H_2_O	60	90	NIA	2.5–10	[25]
MPC	H_2_O	60	5–90	350 ± 50	20	[26]
St	NIA	rt ^4^	270	315–400	NIA	[27]
AA	NIA	rt	270	315–400	NIA	[27]
MeOEGMA	NIA	rt	270	315–400	NIA	[27]
VPA	NIA	rt	270	315–400	NIA	[27]
BA	NIA	rt	270	315–400	NIA	[27]
MTAC	H_2_O	rt	5–90	365	5	[28]
SPMK	H_2_O	rt	5–90	365	5	[28]
AA	H_2_O	NIA	30, 45, 60, 90	350 ± 50	NIA	[29]
MeHA	NIA	NIA	0.5, 1, 1.5, 2, 4	NIA	5	[30]
VSA	H_2_O	rt	40	365	NIA	[31]
VPA	H_2_O	rt	20, 50, 90	365	NIA	[32]
VPA	H_2_O	rt	40	365	NIA	[33]

^1^ Temperature; ^2^ all cited papers were based on the same synthetic procedure; ^3^ no information available; ^4^ synthesis was carried out at room temperature.

**Table 2:** Surface modifications of poly(ether ether ketone) via ATRP method.

Initiator	Monomer	Catalyst Complex	Solvent	T (°C)	Catalyst Concentration		Reference
					ppm (Catalyst/Monomer)	ppm (by Weight)	
2-BiBr	MPS	Cu^II^Cl_2_/BPY	MeOH/H_2_O	rt ^1^	25,071	3533	[58]
2-BiBr	MeOEGMA	Cu^II^Br_2_/BPY	H_2_O	30	2270	2251	[58,59] ^2^
2-BiBr	NIPAM	Cu^II^Br_2_/PMDETA	MeOH/H_2_O	rt	10,000	2753	[58]
2-BiBr	PEGMA	Cu^II^Cl_2_/BPY	H_2_O	30	56,977	6130	[11]

^1^ Synthesis was carried out at room temperature; ^2^ all cited papers were based on the same synthetic procedure.
